# Supplementary material for: Nivolumab plus Ipilimumab versus Existing Immunotherapies in Patients with PD-L1-Positive Advanced Non-Small Cell Lung Cancer: A Systematic Review and Network Meta-Analysis
Source: Cancers (Basel). 2020 Jul 15;12(7):1905. doi: 10.3390/cancers12071905 (PMC7409193; doi:10.3390/cancers12071905)
Supplement: Supplementary file 1 [file cancers-12-01905-s001.pdf]

Supplementary Materials

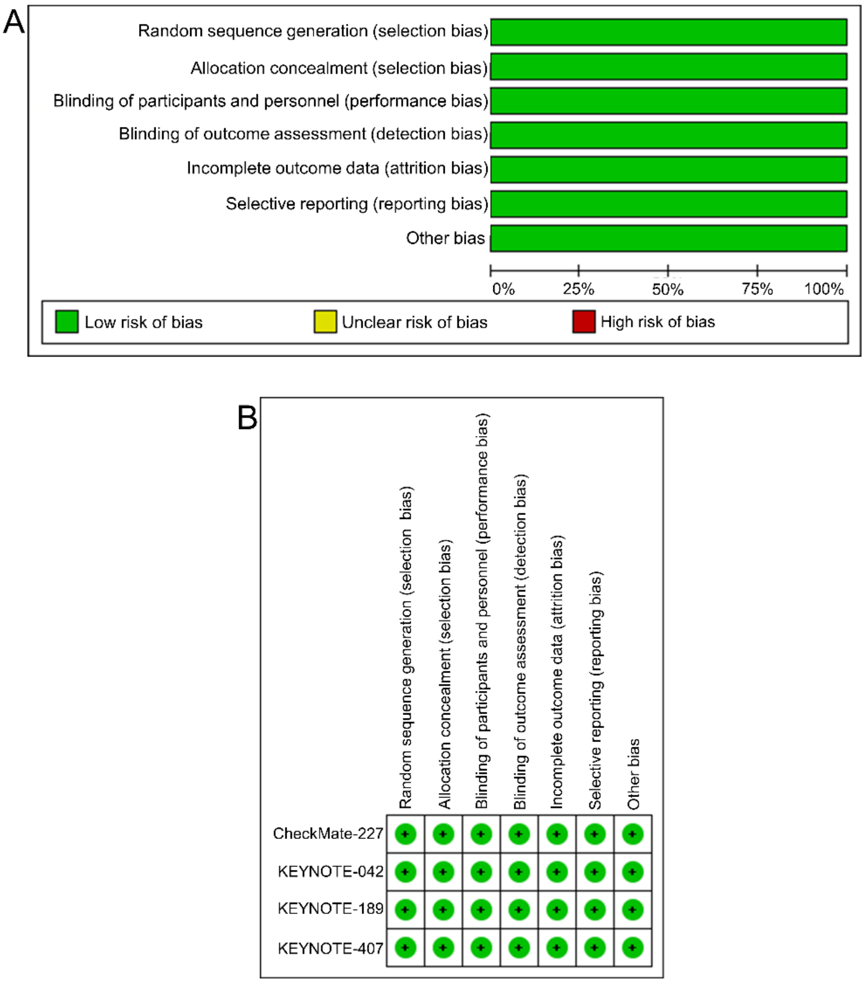

Figure S1: Risk-of-bias graph and summary. (**A**) Risk-of-bias graph: review of judgment in terms of each risk-of-bias item shown as percentages across all included studies. (**B**) Risk-of-bias summary: review of authors' judgments in terms of each risk-of-bias item for each included study.

Table S1: Sensitivity analysis

Treatment comparisons	PFS		OS	
	PD-L1 ≥ 1%	PD-L1 ≥ 50%	PD-L1 ≥ 1%	PD-L1 ≥ 50%
Niv vs. PBC	0.994 (0.798–1.225)	0.787 (0.558–1.079)	0.885 (0.691 1.116)	0.818 (0.567–1.143)
Pem vs. PBC	1.072 (0.943–1.213)	0.814 (0.666–0.984)	0.812 (0.708 0.927)	0.694 (0.560–0.849)
Pem+PBC vs. PBC	0.444 (0.339–0.569)	0.432 (0.259–0.678)	0.477 (0.337 0.654)	0.432 (0.259–0.678)
Niv+Ipi vs. PBC	0.823 (0.708–0.950)	0.625 (0.488–0.788)	0.793 (0.668 0.935)	0.706 (0.547–0.896)
Pem vs. Niv	1.091 (0.845–1.387)	1.064 (0.712–1.530)	0.931 (0.701 1.213)	0.875 (0.570–1.287)
Pem+PBC vs. Niv	0.451 (0.318–0.623)	0.565 (0.301–0.971)	0.547 (0.354 0.806)	0.546 (0.286–0.947)
Niv+Ipi vs. Niv	0.832 (0.710–0.970)	0.805 (0.637–1.005)	0.903 (0.758 1.068)	0.877 (0.678–1.116)
Pem+PBC vs. Pem	0.415 (0.308–0.548)	0.537 (0.308–0.871)	0.590 (0.405 0.830)	0.630 (0.360–1.026)
Niv+Ipi vs. Pem	0.770 (0.632–0.930)	0.775 (0.563–1.042)	0.982 (0.787 1.210)	1.029 (0.736–1.401)
Niv+Ipi vs. Pem+PBC	1.887 (1.385–2.513)	1.536 (0.864–2.533)	1.713 (1.160 2.443)	1.734 (0.972–2.870)

Sensitivity analysis performed excluding KEYNOTE-407[36]. Data are expressed as hazard ratio and 95% credible intervals. PFS, progression free survival; OS, overall survival; PD-L1, programmed cell death ligand 1; Niv, nivolumab; PBC, platinum-based chemotherapy; Pem, pembrolizumab; Ipi, ipilimumab.

Table S2: Sensitivity analysis for ranking assessment

Treatment comparisons	SUCRA for PFS [rank]		SUCRA for OS [rank]	
	**PD-L1 ≥ 1%**	**PD-L1 ≥ 50%**	**PD-L1 ≥ 1%**	**PD-L1 ≥ 50%**
PBC	32.9 [3]	2.1 [5]	3.7 [5]	2.9 [5]
Niv	32.2 [4]	39.3 [3]	31.3 [4]	31.7 [4]
Pem	10.4 [5]	36.2 [4]	53.3 [3]	58.5 [3]
Pem+PBC	100.0 [1]	97.4 [1]	99.9 [1]	98.0 [1]
Niv+Ipi	74.6 [2]	75.1 [2]	61.9 [2]	59.0 [2]

Sensitivity analysis performed excluding KEYNOTE-407[36]. Data are expressed as surface under the cumulative ranking curve (SUCRA) and efficacy rank within each treatment regimen.PFS, progression free survival; OS, overall survival; PD-L1, programmed cell death ligand 1; Niv, nivolumab; PBC, platinum-based chemotherapy; Pem, pembrolizumab; Ipi, ipilimumab.
